# Understanding the Factors and the Corresponding Interactions That Influence Construction Worker Safety Performance from a Competency-Model-Based Perspective: Evidence from Scaffolders in China

**DOI:** 10.3390/ijerph16111885

**Published:** 2019-05-28

**Authors:** Kongzheng Liang, Ivan Wing Hong Fung, Chaohua Xiong, Hanbin Luo

**Affiliations:** 1 Department of Architecture and Civil Engineering, City University of Hong Kong, Hong Kong, China; kongliang2-c@my.cityu.edu.hk (K.L.); ivan.fung@cityu.edu.hk (I.W.H.F.); 2 Department of Construction Management, School of Civil Engineering and Mechanics, Huazhong University of Science and Technology, Wuhan 430074, Hubei, China; luohbcem@hust.edu.cn

**Keywords:** construction safety, construction workers, competency theory, intermediating effects, factor analysis, structural equation modeling

## Abstract

*Purpose:* Construction workers’ reactions to safety-related issues during operation vary from person to person due to their different occupational levels, which can be attributed to various influencing factors and their correspondingly complicated interactions. This research aims to propose an integrated framework to combine the concepts of these factors and provide a holistic interpretation of the interrelationship among them. *Methods:* Based on items that were mainly extracted from competency theory, exploratory factor analysis (EFA) and confirmatory factor analysis (CFA) were conducted to identify the critical factors from the data collected from 243 scaffolders on Wuhan Metro construction sites. The interactions among the identified factors were then analyzed, and the safety competency model was thus established with the use of structural equation modeling (SEM). *Results:* A total of 17 items were identified as critical to workers’ safety competency, and these were further tested and attributed to four factors: (1) individual character and inclination; (2) self-adjustment and adaptability; (3) working attitudes; (4) safety-related operation qualification. Subsequent analysis showed that all the factors significantly contributed to one’s safety competency, and individual character and inclination contributed most to the formation of one’s ability, while the intermediating effects of self-adjustment and adaptability should not be neglected both in theoretical and practical terms. The resultant safety competency model consisting of these four factors was revealed to share a hierarchical structure with the classical competency model. *Significance:* This study provided an integrated theoretical framework and a set of modeling approaches to combine the related concepts and facilitate a greater understanding of construction safety in terms of workers’ characteristics and behaviors. *Practical implications:* This study presented a tentative approach for assessing construction workers’ safety competency, as well as emphasized to the managers and professionals the necessity of developing training systems to ensure workers are integrated into a crew in an appropriate and smooth manner. *Limitations and Future Work:* The volume and the scope of samples impeded the study from achieving a more generalized result and a more cost-efficient data collection approach is in need of development for a comprehensive and in-depth investigation.

## 1. Introduction

The construction industry is widely known as one of the most dangerous engineering industries. Research from many nations has shown that construction workers still suffer from a higher proportion of occupational injuries and fatalities compared with other industries [1,2,3]. Owing to the numerous construction projects that are being carried out due to fast urbanization, this problem can be especially acute in developing countries, for example, China. According to the report of the State Council Security Committee of Chin, in the first half of 2018, there were 1732 workplace accidents resulting in 1752 deaths in the construction industry, with a year-on-year increase of 7.8% and 1.4%, respectively. The total number of accidents in the construction industry has ranked highest out of all industries for nine consecutive years so far, in addition to the year-on-year increase that has been observed since 2016. 

Meanwhile, the rapid development of the construction industry in China has resulted in the employment of a considerable number of migrant construction workers at varying occupational levels [4], resulting in different performances at work when confronting the similar issues. Such differences will lead to a severe consequence for workers when it comes to safety-related issues during operation, as people are prone to overestimate their ability to handle risks [5,6] and thus unsafe behaviors are adopted, which is generally accepted as the fundamental cause for most accidents [7,8,9]. Therefore, for reducing the occurrence of construction accidents, it is essential to assess the influencing factors that affect workers’ safety performance at work. 

Previous studies have evaluated different factors which influence workers’ safety performance, and an emerging trend is seen in which researchers are prone to applying a systematic analysis approach to include more factors into the assessment models [10,11,12]. However, most factors were merely selected from previous studies without a holistic guideline. Hence, the factor selection process depended on the authors’ different preferences for articles and was influenced by their prior cognitions on the constitution of the models, which may result in a non-objective model. Even if the identified factors shared similar connotations, various terms and definitions from different research contexts also presented barriers to understanding readily. In other words, not only the systematic analysis techniques but also an integrated theoretical framework is essential for the generalization and feasibility of the assessment models against workers. 

The competency theory may be a reasonable solution when addressing the selection of factors and the integration of concepts [13]. The application of the competency theory has brought about the improvement in occupational education, training, and assessment in many jobs [14], including the a few non-management-level jobs [15,16]. Therefore, taking scaffolders as an example, this paper aims to present a systematic hybrid approach incorporating the competency theory and systematic analysis methods (i.e., factor analysis and structural equation modeling) to identify the critical factors required for a scaffolder to safely complete operation tasks, and to measure how much the effects of the identified factors contribute to workers’ safety competency. From a practical perspective, the prospective results have great potential in optimizing assessment and training for construction workers.

## 2. Literature Review

The correct estimation about how well a worker can deal with uncertain situations is not an easy task as various influencing factors are involved in this process. For instance, Abdelhamid proposed that fatigue and distraction caused by a severe workload on sites negatively affect workers’ actions, thus increasing the risks that confront builders in the work place [17]. The findings of Choudhry and Fang indicated that workers were involved in unsafe conditions because of a lack of safety awareness and risky attempts to prove to their coworkers that they were “tough guys” [8]. Some studies implied that workers’ incorrect risk assessment of the probabilities and consequences of their decisions and behaviors also may push them into hazardous situations [18,19]. It is summarized that most of the current studies tend to divide the influencing factors into two types, namely, internal factors that refer to individual personalities and external factors relating to the influence of the working environment, such as job stress and organization culture. 

Furthermore, the estimation of how well a worker can deal with uncertain situation is expected to be more complicated when taking accounts the interrelationships among these influencing factors. In response, although the terms and specific definitions used may vary from different studies, many researchers have contributed their thoughts to this issue, and the importance of systematic thinking towards these factors and their interactions has been unanimously confirmed. Shin et al. applied a system dynamics approach to explicitly represent workers’ mental process from perceiving risks to adjusting safety attitudes, and then taking actions [6]. The systematic method of structural equation modeling (SEM) was generally proved to be effective in identifying critical factors and key paths that pose an impact on construction workers’ risk tolerances [10]. The relationship among the organizational factors of safety culture and safety climate with safety behavior was highlighted by a systematical model focusing on temporary construction workers [11]. Zhang et al. provided insights into cause–effect relationships among the workers’ perceived influential factors and goals in safety behaviors with a mixed systematic approach [12]. However, owing to the fact that the influencing factors were mainly selected from previous studies conducted under different contexts, it is challenging to combine all the concepts discussed into an integrated theoretical framework, thus placing an obstacle in the way of obtaining a better systematic understanding towards these factors and their interactions.

In our study, the competency theory served as a theoretical foundation for offering a more comprehensive and understandable framework to integrate the relevant concepts and facilitate understanding. Since the competency theory was proposed by White in 1959, this idea has received extensive attention immediately and been developed continuously by researchers in different fields. The competency was initially defined as the individual’s capacity to interact effectively with their environment, an ability attained through prolonged feats of learning [20], and McClelland further enriched the connotation of this concept by pointing out that comprehensive elements such as learning ability, communication skills, leadership, and general problem-solving abilities should be embedded [21]. Afterward, following the exploration by MeLagan in which the competency theory was applied to model the capability of specific jobs [22], the competency study was further advanced by Boyatzis’s work on developing tailored competency interventions to suit unique characteristics of an organization [23]. 

Based on these previous studies, Lyle and Signe Spencer’s work made a remarkable contribution to competency research and application [14]. Particularly, they developed generic competency models for more than 200 jobs, and the resultant competency dictionary, including more than 20 sets of generalized attributes, was expected to give a reasonable explanation to 80% or more ratios of behavior and the resulting consequences in each model [24]. These attributes then led to a systematical illustration known as the “Iceberg Model” to interpret the connotations and structure of competency at work [13]. In this regard, the competency theory is qualified as a source for identifying factors, and to offer an incorporated framework for understanding the interactions within.

Inspired by its successful applications in many domains [25], many researchers contributed their efforts to develop context-specific competency models for the construction industry. Among them, the competency of project managers was the most intensively discussed topic due to the critical role they play in determining the success of a project [26,27,28]. Other emerging investigation interests within the industry were identified such as cost estimators [29], construction supervisors [30] and architects [31]. Even for the studies that focused on the safety-related competencies, researchers also tended to pay attention to the management staff [32,33]. However, on the other hand, although the frontline workers are in the most hazardous position in the industry, only rarely have competency studies been conducted to shed light on their jobs. 

As a result, the concepts and terms adopted in this article are derived from Lyle and Signe Spencer’s work. For example, most of the alternative items belonging to one factor come from the generic competency dictionary [34,35], the identified factor structure is expected to comply with the Spencers’ iceberg model, and these factors should differ in the extent to which they can be taught [14]. The factors at the upper levels were more observable and susceptible, while those hidden in the lower levels were difficult to measure or train but usually have a larger influence on workers’ performance. Given the very nature of scaffolding operation and construction workers, the management-level-oriented items were not included in the list. Furthermore, due to focusing on the critical factors that affect safe working procedures, the term of “safety competency” is adopted to represent the safety-related aspect of individual competency and is defined as “The underlying characteristics of a construction worker that are causally related to criterion-referenced safe performance during daily operation”, which is also based on Spencer’s work.

## 3. Methods and Materials

This research is comprised of three stages: (1) questionnaire survey, (2) factor analysis, and (3) modeling of safety competency. The details of these parts are presented as follows.

### 3.1. Questionnaire Survey 

The questionnaire survey occurred in two phases: a pilot survey and a large-scale survey. Potential items of competency factors were gathered from different resources to deliver a formal questionnaire, and then a large-scale questionnaire survey was conducted to capture the workers’ perceptions on these items. 

#### 3.1.1. Pilot Survey

During the pilot survey, possible items of safety competency factors were collected from the following resources:

- As the primary source, the general competency dictionary [34,35] was investigated and refined to capture the potentially applicable items. Notably, given the very nature of scaffolding operation and construction workers, most of the management-level-oriented items (e.g., leadership, strategic decision making) were not extracted.
- The interview results are another leading resource. A structural interview method called the behavioral event interview was also implemented among 11 seasoned workers with decent safety records. In this method, the interviewee was asked to describe his/her real experience during operation from several aspects including occurrence situation, work tasks, workers’ action, and the resulting consequences. The results were summarized and refined in order to identify some shared opinions regarding influencing items [14].
- The national occupational skill standards of scaffolding workers (NOSSS) and analysis reports of previous accidents were introduced as a supplementary reference for the job requirements of scaffolding workers.

Consequently, a primary list that contains all the potential items of competency factors was generated, serving as a basis for the subsequent formation of the questionnaire.

#### 3.1.2. Large-Scale Survey

Before designing the questionnaire, all items measured in the pilot survey were filtered and summarized by comparing the core connotations of each item, mainly according to the definitions and key actions described in the general competency dictionary. Then, through a discussion with two crew leaders and two seasoned worker, the description towards each item was more intelligible for workers, in terms of contents and expression style.

Two kinds of questions were designed given the different implications of the remaining items. For the items that can be observed or perceived easily, taking the physical condition (Item 1) as an example, the description is stated as “I am energetic and strong enough to handle my daily work, even for the overtime task.” and the answer would be to what extent their own condition can fit the statement. As for the unobservable items, for example, the item regarding steadiness (Item 13), the statements will be changed to “Displaying the courage during daily operation is not an appropriate way to earn other worker’s respect”. Moreover, respondents were asked to grade the item depending on how much they agree with such an assertion. Notably, for the items that may play an intermediating role between the individual and organization, coworkers’ attitudes will be considered in the statement of questions, such as the item about the working relationship (Item 9), which was described as “My colleagues are willing to seek my advice, and I am able to address the conflicts within the crew without help from crew leaders”. 

The questionnaire was distributed to scaffolders to collect their perception of the items measured by Likert scales. For a more straightforward demonstration of workers’ propensity on these items, a four-point Likert-type scale without neutral options was adapted and arranged as four levels, corresponding to respondents’ perception on the statement for each item, namely, 1 (i.e., strongly disagree with this assertion/my condition does not fit this statement at all), 2 (i.e., disagree with the assertion/my condition partially fits this statement), 3 (i.e., agree with the assertion/my condition mostly fits this statement) and 4 (i.e., strongly agree with the assertion/my condition totally fits this statement mostly). More details of questionnaire items can be seen in Appendix A.

### 3.2. Factor Analysis 

After data collection, the factor analysis was performed to determine the critical items which influence a worker’s safety performance. This section included the explanatory factor analysis (EFA) and the confirmatory factor analysis (CFA), and accordingly, the raw data were separated randomly into two sets with the same number of samples (i.e., calibration samples vs. validation samples), serving to group the potential critical items and test processes, respectively:

- Factor analysis started with the EFA on the results of the questionnaire survey. The EFA aims to use a smaller number of dimensions to render the original data structure, and hence exploring the structure that underlies the gathered data [12]. Specifically, appropriate numbers of primary structural factors (i.e., the numbers of groups) were determined according to the concentration degree of listed items, and all items were thus assigned to different groups by weighing their varying loadings on each primary structural factor.
- The CFA was conducted to verify the reliability and validity of the grouping results. In contrast with EFA, CFA hypothesizes the numbers of primary structural factors in advance, whether or not these factors are correlated [36]. Cronbach’s α value was firstly used to confirm the reliability which determined whether the proposed questionnaire was capable of measuring the identified factors with stable performance. Subsequently, convergent validity and discriminant validity were examined to ensure a strong correlation among items belonging to one factor, as well as an adequate distinction between different factors. Such verification was processed by comparing the standardized factor loadings, constructing the reliability and average variance extracted with the standard criterion.

The factor analysis results also served as a basis for the construction of the measurement model in the subsequent SEM analysis. The measurement model is concerned with how a latent issue is conceptualized and measured by manifest variables. It turned out that the factor analysis offered a reliable tool for revealing the constructs underlying the measuring indicators, especially for the condition that the prior knowledge regarding factors or patterns of measuring indicators is insufficient [37,38]. 

### 3.3. Modeling of the Safety Competency for Scaffolding Workers

The SEM is applied to quantitatively profile scaffolders’ safety competency due to its superior performance in handling the complex relationships among factors and estimating the interactions involved [39]. A structural equation model comprises of a measurement model and a structural model, which respectively aims to characterize the latent factors qualitatively and analyze their interactions quantitatively. 

Based on the measurement model obtained from the factor analysis, the interactions among latent factors (i.e., those mentioned above as “structural factors”) were hypothesized, followed by a test on the regression estimations yielded from the structural model. Finally, after a test of the goodness-of-fit of the proposed model, the key influential path between latent factors and safety competency was identified, and thus a safety competency model for scaffolding workers was established.

Meanwhile, as a theory-driven method, all the findings obtained from SEM should be supported by theory. In other words, in addition to meeting the statistical standards, following the prior theory is also necessary so that the relationships discovered during the SEM process can be interpreted from a practical perspective. This issue was considered and is reflected by the model modification process described in later sections. 

### 3.4. Survey Samples

In order to mitigate the stress from increasing traffic congestion and population, the city of Wuhan is engaged in a large-scale metro construction, thus providing sufficient sources for our investigation. On the other hand, the scaffolding workers were chosen as respondents due to the hazardous nature of their high-altitude work. Among the six construction sites surveyed, the number of scaffolders at each work site varied between 20 to 50. Construction site worker safety performance estimates were mainly based on these regulations: Standard for Construction Safety Assessment of Metro Engineering (GB 50715-2011) and Quality and Safety Check Points of Urban Rail Transit Engineering (2011). 

After 15 weeks of investigation including an interview and questionnaire survey, 243 scaffolders from six construction sites of the Wuhan Metro 3rd line project participated in the survey, and 205 responses were received, which has reached the standard of using SEM for empirical research [40]. Table 1 presents the profile of valid respondents. All subjects gave their informed consent for inclusion before they participated in the study. In addition, the individual information of respondents in our study will be highly respected and strictly confidential. 

## 4. Modeling

### 4.1. Identification of Critical Items

The results demonstrated that 62 potential items that may influence the safety competency were obtained from the pilot survey (see Table 2). 

Based on the description of the definitions and key actions, 19 items relating to physical tasks were collected from the generic competency dictionary. Furthermore, 15 items from the behavioral event interview were denoted by the respondents’ oral description. Accident reports provided 13 items which were summarized as one of the accident causes in the original documents. A total of 15 items were extracted from the NOSSS, most of which were described as the occupational ethics in the book. 

Then, items with similar meanings were filtered and summarized, followed by a discussion with four crew leaders and seasoned workers for a more transparent and understandable questionnaire for the workers. Most of the definitions were given with reference to the generic competency dictionary [14,35]. As for the items that cannot be directly attributed to one single attribute in the dictionary but were frequently mentioned by other resources, a new term was created to cover its core ideas, such as “Upholding the Principle”,” Prudence” and “Steadiness”. Additionally, the legal common sense required by the national standards was also added in the final list, although no other resources mentioned this item during the survey. 

After analyzing the results of the pilot survey, a list of 19 potential critical items was prepared for the large-scale questionnaire survey, as well as the corresponding detailed definitions (see Table 3). Consequently, the large-scale survey showed that 18 items among the original 19 items were determined as the critical ones, while the item of legal common sense was excluded due to its low mean value (see Table 4).

### 4.2. Factor Analysis

#### 4.2.1. Explanatory Factor Analysis

Generally, factor analysis started with explanatory factor analysis. Explanatory factor analysis aims to reduce variables into several primary components, thus revealing the underlying structure and interrelationship among a number of factors. 

The suitability of conducting factor analysis towards this dataset is evaluated using the Kaiser-Mayer-Olkin sampling adequacy test (KMO’s test) and Bartlett’s Test of Sphericity (Bartlett’s test). The value of the KMO’s test was 0.786, that met the general standard [42], and the Bartlett’s test indicated that the null hypothesis can be rejected with the value of 960.358 (*p* < 0.001). As a result, it turned out that significant interactions exist among factors (see Table 5).

Then, principal component analysis was applied to extract the items with the initial eigenvalues and factor loading set to greater than 1.0 and greater than 0.4, respectively. After removing the items with invalid or excessive loadings (i.e., I12 Calmness), a four-factor solution with 17 items accounting for 66.021% of the total variance was generated. 

In essence, the factor analysis is a method which replaces a large number of variables with several common factors, with an aim to represent the information contained in these variables to the maximum extent. Thus, the concepts of factor loadings and explained variance are derived. The factor loading is defined as the correlation coefficient between a variable and a common factor, denoting how well a common factor can elaborate a manifest variable. While the sum of squared factor loadings for all variables for a given factor refers to the percentage of variances in all variables explained by that factor. It means that the grouping results of items (i.e., manifest variables) are determined by their different factor loadings, and the interpretability of the proposed factor structure can be measured by the proportion of the variances explained by factors.

The factor load matrix after rotation was illustrated in Table 6, factor loadings of 17 items corresponding to their common factors were above 0.70, ensuring satisfactory interpretability of common factors [10]. According to the implications of items and the grouping result, these groups were identified as four factors to represent different aspects of the safety competency derived from the competency theory, as shown below.

Factor 1 includes five items, namely, item I15 Stress Tolerance, item I13 Steadiness, item I8 Upholding the Principle, item I2 Professional Integrity, and item I10 Prudence, which accounts for 22.003% of the total variance and is labeled as “individual character and inclination”.

Factor 2 includes six items, namely, item I4 Responsibility, item I7 Time management, item I6 Teamwork, item I5 Applied Learning, item I9 Positive Working Relationship, and item I11 Adaptability, which accounts for 21.271% of the total variance and is labeled as “self-adjustment and adaptability”.

Factor 3 includes three items, namely, item I19 Impact, item I18 Compliance to Procedure, and item I17 Quality Orientation, which accounts for 11.443% of the total variance and is labeled as “working attitudes”.

Factor 4 includes three items, namely, item I14 Safety Awareness, item I3 Technical knowledge and skills, and item I1 Physical Condition, which accounts for 11.305% of the total variance and is labeled as “safety-related operation qualification”.

#### 4.2.2. Confirmatory Factor Analysis

Afterward, CFA was applied to test the reliability and validity of the proposed grouping. Firstly, with regard to the reliability, the Cronbach’s α test was used to evaluate the internal consistency reliability which reflects correlations between questionnaire items belonging to one dimension [43]. This result determines whether the proposed questionnaire is capable of measuring the identified factors with a stable performance. Some professionals, as a rule of thumb, require reliability of 0.70 or higher as a desirable level, while 0.60 was generally accepted as the lowest acceptable threshold [43]. Cronbach’s α values are shown in Table 7; the overall α values indicated that the developed measurement scales were reliable, which means that the grouping of extracted factors is appropriate.

In terms of validity, two types of validities, namely, convergent validity and discriminant validity, are introduced to ensure the strong correlation among items belonging to one factor, as well as the adequate distinction between different factors [11,44]. Validity was verified by standardized factor loadings (FL > 0.5), construct reliability (CR > 0.7), average variance extracted (AVE > 0.5) and the square roots of AVE (larger than the correlation coefficients between factors [45,46,47]. The results listed in Table 8 and Table 9 confirmed the validity of the proposed grouping.

### 4.3. Structural Equation Modeling 

**Figure 1:**
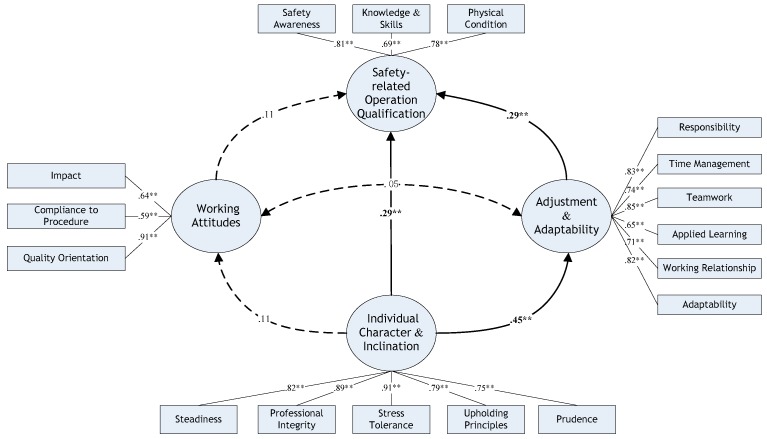
The Hierarchical Model of Construction Workers’ Safety competency. Asterisks accompanying the coefficients indicate the significance level for each path in the figure (i.e., two asterisks indicate *p* < 0.01 and one asterisk indicates *p* < 0.05).

Results of EFA and CFA supported the rationality of grouping these 17 items into four groups. Owing to the fact that the competency theory underpins the design of the questionnaire, there may also be a hierarchy structure among these four groups. Based on the composition of each group and the corresponding definition of items therein, a hierarchical four-factor model was initially established, and the interrelationships among them are shown in Figure 1. 

According to Spencer’s competency model [14], various competency characteristics can be categorized based on the visibility and difficulty level in the training. The characteristics that are located closer to the bottom of the model structure are more likely to become hidden and difficult to develop. For instance, as exterior characteristics, knowledge and skills tend to be visible and measurable, while one’s motives and traits are deeper and more central to personality. Furthermore, between the acquired characteristics and inherent characteristics, the self-concept characteristics are prone to playing an intermediating role at the middle level [16]. Thus, the following hypotheses (H1–H6) were developed to examine the interrelationships among factors:

**Hypothesis** **1 (H1).** *Workers’ individual character and inclination (Factor 1) positively affect their self-adjustment and adaptability (Factor 2).*

**Hypothesis** **2 (H2).** *Workers’ individual character and inclination (Factor 1) positively affect their working attitudes (Factor 3).*

**Hypothesis** **3 (H3).** *Workers’ individual character and inclination (Factor 1) positively affect their safety-related operation qualification (Factor 4).*

**Hypothesis** **4 (H4).** *Workers’ self-adjustment and adaptability (Factor 2) positively affect their safety-related operation qualification (Factor 4).*

**Hypothesis** **5 (H5).** *Workers’ working attitudes (Factor 3) positively affect their safety-related operation qualification (Factor 4).*

**Hypothesis** **6 (H6).** *Workers’ self-adjustment and adaptability (Factor 2) positively correlate with their working attitudes (Factor 3).*

The precondition of conducting an SEM analysis lies in the construction of the measurement model. As mentioned above, the analysis results derived from EFA and CFA at preceding sections provided the measurement model, reflecting the relationships between latent factors and their corresponding measuring indicators. Therefore, there were four latent factors and 17 corresponding measuring indicators (i.e., the aforementioned “items”) involved in the subsequent SEM analysis. For instance, three measuring indicators (i.e., impact, compliance with procedure, and quality-orientation) were included in the latent factor F3 for reflecting one’s working attitudes during operation. Other latent factors and measuring indicators can be processed by analogy. 

Hypothesis testing is the primary step in an SEM analysis. Figure 1 and Table 10 demonstrated the correlation among latent factors and the hypothesis testing results in detail. Solid lines represent important links, and the dashed lines mean the path failed to pass the hypothesis tests. The one-way arrow means that the factor at the beginning is expected to pose an impact on the factor at the end, while the two-way arrow implies that two factors that are connected are more likely to be correlated. The parameters on arrows are the path coefficients, referring to the effects of variables on each other in a structural model, and asterisks accompanying the coefficients indicate the significance level for each path (i.e., two asterisks indicate *p* < 0.01 and one asterisk indicates *p* < 0.05). The results of the original hierarchical four-factor model supported Hypotheses H1, H3, and H4, which were confirmed, while the hypotheses related to factor F3 working attitudes (H2, H5, H6) were all rejected due to the insignificant regression coefficients.

Furthermore, in an SEM analysis, the goodness-of-fit of a model also needs to satisfy a set of criteria including various indicators to guarantee the proposed model can be used for further analysis [12]. These indicators can be classified in three aspects: absolute fit indices, parsimony fit indices, and incremental fit indices [48]. Absolute fit indices provide the most fundamental indication of how well the proposed theory fits the data [49], including the chi-squared test $\left(\chi^{2}\right)$, normed chi-square test $\left(\chi^{2}/\mathrm{df}\right)$, root mean square error of approximation (RMSEA), goodness-of-fit statistic (GFI), and the adjusted goodness-of-fit statistic (AGFI). Parsimony fit indices were developed to seriously penalize for complex models, thus avoiding the selection of a saturated but less rigorous theoretical model that was yielded barely based on the ordinary fit indices [50]. Included in this category are the parsimony goodness-of-fit index (PGFI) and the parsimonious normed fit index (PNFI). Incremental fit indices are a group of indices that measure the improvement status between the proposed model and a baseline model. For these models, the null hypothesis is that all variables are uncorrelated [49]. The comparative fit index (CFI), normed fit index (NFI) and non-normed fit index (NNFI) are typical examples belonging to this type of indice. The results of goodness-of-fit testing are listed in Table 11, which revealed that the proposed model is basically qualified for fitting the data.

However, as stated by Kendall and Stuart, “A statistical relationship, however strong and however suggestive, can never establish a causal connection: our ideas on causation must come from outside statistics, ultimately from some theory or other [51].” The meaningful relation between variables is determined by no statistical relationships but logical deduction. Given the actual meaning of factor F3 working attitudes, as well as the relatively unsatisfactory condition of goodness-of-fit testing results, it is not reasonable to exclude this factor out of the safety competency model merely due to its relatively ambiguous correlations with others. Therefore, further analysis was conducted to examine whether there exists a higher-level factor structure which can provide a more comprehensive reflection on one’s safety competency, and thus a deep insight into the potential impact from working attitudes is obtained. 

For the higher-level concepts, two performance-related indicators were introduced to demonstrate one’s overall safety competency, namely, safety-related behavior and safety-related test performance. Safety-related behavior refers to the safety index, an indicator which has been adopted in previous studies for measuring safety performance from the perspective of risky actions during operation [52,53]. Likewise, the outcomes of these two processes were also transformed into four levels in order to comply with the evaluation scales used in the above questionnaire survey.

From the data sources of Standard for Construction Safety Assessment of Metro Engineering (GB 50715-2011) and Quality and Safety Check Points of Urban Rail Transit Engineering (2011), a total of six unsafe behaviors displayed by scaffolders were identified and classified including: (1) scaffolders worked in sleety weather without wearing non-slip shoes, (2) scaffolders worked at heights of more than 2 m without wearing a harness, (3) scaffolders climbed up and down the scaffold without protection, (4) scaffolders stood in a location without a sufficient scaffolding floor, (5) scaffolders threw member bars from heights in the process of dismantling, and (6) scaffolders stacked too much material on the scaffolds. After determining the unsafe behavior list, we used video surveillance to observe the behaviors of the scaffolders. In order to achieve further quantification of the scaffolder behavior results, each record of unsafe behavior was converted to a percentage. The safety index (SI) [52] was adopted to test the unsafe behavior using the following formula:

(1) $$SI=\frac{N_{1}}{N_{1}+N_{2}}$$

where *N_2_* is the number of observed instances of safe behavior, *N_1_* is the number of observed instances of unsafe behavior, and *N_1_* + *N_2_* is the sum of all instances of the previously specified safety-related behavior.

Moreover, the workers’ scores on a test regarding the identification of hazards on sites were used as the indicator “Safety-Related Test Performance,” and were provided by a tentative training system applied in the Wuhan Metro Construction [54,55]. During the test, this system displayed worksite photos to workers and required them to point out the hazardous locations and dangerous behaviors within a given period. The test score for each participant was rated according to the accuracy of the answers and his/her response time. 

**Figure 2:**
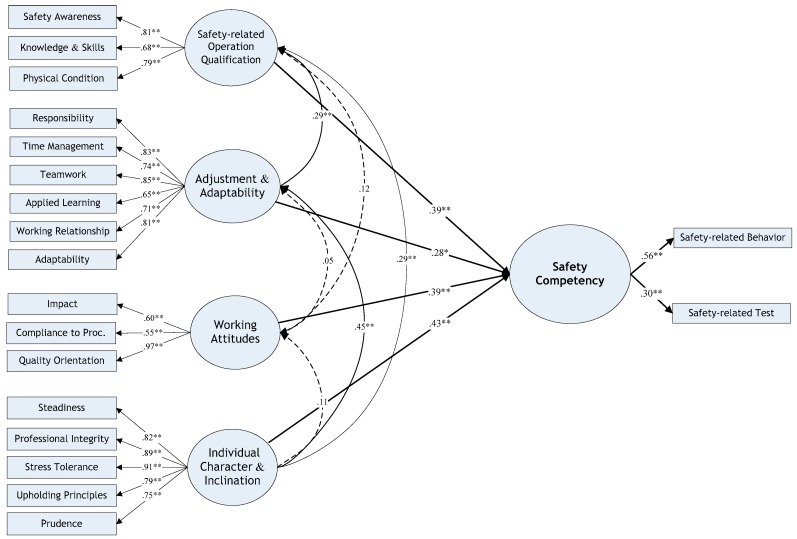
The Modified Hierarchical Model of Construction Workers’ Safety competency. Asterisks accompanying the coefficients indicate the significance level for each path (i.e., two asterisks indicate *p* < 0.01 and one asterisk indicates *p* < 0.05).

Figure 2 illustrated the modified hierarchical four-factor model. It turned out that four latent factors all demonstrated a significant influence in this higher-level conceptual factor structure, including factor F4 working attitudes. Similarly, the assessment of the model fit was also conducted against this model, resulting in a superior result in terms of all the fitness indices. More details are shown in Table 11. The differences between the two models supported the assumption regarding the impact of F3, and also to some extent, implied the existence of a superior concept which is more capable of interpreting the latent and common properties among latent factors. Moreover, the resultant additional hypotheses were thus developed, as shown below. All testing results for the modified model supported the existence of the higher-level concept, and details can be seen in Table 10:

**Hypothesis** **7 (H7).** *Workers’ individual character and inclination (Factor 1) positively affect their safety performance.*

**Hypothesis** **8 (H8).** *Workers’ self-adjustment and adaptability (Factor 2) positively affect their safety performance.*

**Hypothesis** **9 (H9).** *Workers’ working attitudes (Factor 3) positively affect their safety performance.*

**Hypothesis** **10 (H10).** *Workers’ safety-related operation qualification (Factor 4) positively affects their safety performance.*

The SEM analysis ended with the identification of the key influencing paths. Both direct and indirect effects from latent factors on the safety competency were examined to yield a better understanding of the formation of one’s safety competency. The results are stated in Table 12. The factor F1 individual character and inclination contribute the most to safety competency both in terms of direct effects and indirect effects. Safety-related operation qualification and working attitudes have a similar direct effect on safety competency, and the cumulative effect on safety competency from self-adjustment and adaptability also achieved the equivalent level with the aid of an intermediating effect posed by the factor F4 safety-related operation qualification.

## 5. Discussion

### 5.1. The Constitution of Critical Items

Nineteen of the original safety competency items identified and defined were mainly derived from the competency dictionary. As a result, although all items were modified based on various reference resources including previous studies, accident records, and interviews, the results of the identification of critical items showed a more worker-oriented tendency compared with the previous research focusing on this area [10,11,12,56,57]. The external impact or organizational impact was highlighted in these studies, while this paper takes these issues into account through by investigating the workers’ perception towards the influence from their working circumstances, which is also in line with the basic definition of the competency theory. That is to say, the proposed model paid more attention to a worker’s reaction towards the changes in his/her working environment instead of the impact of these external changes. 

Another difference from previous research lies in the composition of selected items. More items regarding operation stages were identified, and therefore, a physical labor-related trait emerged. Specifically, in comparison with the ability model focusing on the managers on sites, the proposed model is mainly characterized by requirements on harmonious cooperation with workmates and the compliance with regulations, instead of control, decision, and organization [26,58]. While the construction work characteristics, such as heavy manual work and informal organization [59], were somewhat outlined by the survey, results of the highest grade were observed for the physical conditions (2.985) and results of the lowest grade was found for legal-related common sense (1.434). 

### 5.2. The Structure of Latent Factors 

EFA and CFA attributed the critical items into four latent factors mathematically, and the practical implications of such a factor structure were further given with the combination of SEM and the competency theory. In particular, these four factors, respectively, represent different levels in the classical competency hierarchy framework proposed by Spencer [14]. Factor 2 served as the foundation of the model since individual character and inclination were formed at one’s early age, indicating that this feature is not easily influenced by the external environment. Factor 2 and Factor 3 constituted the middle levels of the hierarchy as these features were formed in the socialization process of human beings, reflecting one’s opinions on how to deal with the requirements from his/her organization, both in terms of working and communicating. The most exterior factor is Factor 4 since it is the latest-formed feature, which also means that it is the easiest to observe and interfere with. In general, the factors that lie in the interior level are supposed to pose an impact on the exterior factors. In other words, workers’ inherent personalities will determine their attitudes and reactions towards the external circumstances, and subsequently affect the skills and behavior that they performed at work.

Moreover, even though the reliability and validity of the grouping of critical items have been confirmed, it is noteworthy that there was a disparity between the workers’ perceptions on different factors and the variances that each latent factor could account for. Workers’ perceptions on these items were reflected in the mean values in Table 4, implying that the workers tended to highlight the characteristics at the surface level but underestimate the importance of individual personality (i.e., factor F1 individual character and inclination) to their daily work, and a similar phenomenon also can be observed in factor F3, working attitudes. This may be interpreted as the result of the differences between characteristics at different levels. The influence of characteristics at the bottom of the structure is difficult to directly perceive in ordinary workers due to their invisibility. On the other hand, the higher mean values for factor F4, safety-related operation qualification also supported such an inference. Workers are used to paying more attention to characteristics that they have a direct and obvious interest in, and naturally, the visible characteristics such as physical condition and operation skills are more likely to be the recruitment requirements proposed by employers [14]. 

### 5.3. Significant Influencing Factors and Paths

The hierarchical model and the modified model were validated to profile the potential structure among the factors contributing to safety competency. It turned out that the proposed models satisfied general goodness-of-fit values. The leading factors that influence one’s safety competency and relevant performance can thus be identified via direct and indirect effects of safety-related operation qualification, working attitudes, self-adjustment, and adaptability, as well as individual character and inclination. In this study, individual characters and inclinations exert the most significant influence on safety competency both in terms of direct and indirect effects. However, as mentioned before, it is difficult to modify one’s inherent characteristics, which means that the intermediating factors should be highlighted in practical terms. 

Regarding other factors, it is not surprising that the relatively visible factors (i.e., safety-related operation qualification, working attitudes) also play a critical role in developing safety competency as most current training programs and on-site regulations were aimed at the enhancement from these two perspectives [60,61,62]. It is noteworthy that the significant paths between intermediating factors (i.e., self-adjustment and adaptability) and the other two factors also proved the effectiveness of introducing the competency theory. In other words, the significant influence from self-adjustment and adaptability reminds the researchers that successful communication and cooperation among coworkers is necessary for developing workers’ safety competency, especially considering that few efforts have been devoted to teaching and training workers how to integrate into a crew appropriately [63,64]. 

### 5.4. Implications

The application of the competency model into the construction worker safety literature provides the researchers with a common basis to discuss the scattered concepts related to worker safety. Critical influencing factors identified in the previous research such as safety culture, organization climate, and safety management [10,11,12] were somehow manifested with the languages from the competency theory, thus making the safety-related job requirements for a construction worker more interpretable and accessible. The incorporation between the competency theory and the SEM technique extended the implications of the relationships among factors beyond the statistically estimated values, which is instructive for alleviating the difficulties in interpreting the SEM results [65]. 

Findings supported the existence of the hierarchical structure underlying influencing factors derived from the competency theory, and Spencer’s model profiled a plausibly holistic illustration for the interactions among these factors. By combining the prior theory with the systematic analysis technique, this exploration thus yielded a more integrated and interpretable framework to facilitate the understanding of the reasons behind workers’ different responses when confronting the same safety-related issues. Furthermore, additional similar theories in areas of human resources are expected to be tested or developed by the issues in the construction industry, which may provide an opportunity for researchers from both sides to extend the boundaries of knowledge. 

There are also practical implications of the findings. Firstly, as the proposed model revealed some hidden characteristics for safely conducting scaffolding work, it may be possible for the managerial staff to form specific and cost-efficient training strategies to reduce on-site injury and incidents [32]. For human resource personnel, the competency model is also useful as an assessment tool for hiring and selecting tasks [66]. On the one hand, for the visible factors, continuous training is not only useful but also necessary given their accompanying susceptibility. On the other hand, some critical factors are difficult to develop through general training but still have a profound impact on workers’ performance. This is when the tailored strategies should be implemented to manage such impacts or even filter the incompetent workers. 

Another noteworthy practical implication lies in the findings referring to the intermediating role of the factors at the middle level of the proposed competency model. In addition to the usual criteria, such as physical condition, skills, and knowledge, this study also highlighted the importance of one’s organizational capabilities (e.g., adjustment and adaptability) as a job requirement for scaffolding workers. As a result, application of the competency theory revealed a potential blank in occupational training for construction workers [64]. Namely, it would help to reduce risky actions and injuries if a training project were developed that taught the workers how to adjust themselves to fit into a crew and how to communicate with others during daily work.

## 6. Conclusions

Whether a construction worker can safely complete operation tasks is influenced and determined by various characteristics. A systematic and comprehensive understanding of these characteristics is important for improving the workers’ safety performance and preventing construction accidents. Based on the competency theory, with the incorporation of EFA, CFA, and SEM, this paper established a model for outlining and assessing a construction worker’s safety competency. It was found that four factors significantly contributed to a worker’s safety performance, namely, safety-related operation qualification, working attitudes, self-adjustment and adaptability, as well as their individual character and inclination. 

Furthermore, the proposed model followed the fundamental structure of the classical competency model. Individual character and inclination contributed most to the formation of one’s ability, while this factor cannot be perceived or observed directly by workers in their daily work, as well as taught or modified easily. On the other hand, other important influence resources included safety-related operation qualification and working attitudes, which are related to the measurable indicators such as skills, physical condition, and compliance with the regulations, and certainly most training and management tools were focused on such attributes. Between these two constructs, self-adjustment and adaptability played an intermediating role to link the workers’ internal traits to the external working environment. However, regardless of the significant influence of this factor, little training work has been done to optimize workers safety competency in this respect.

To the best of the authors’ knowledge, this study is one of the first attempts regarding frontline construction workers referring to the competency theory and methods. The aim of this study is to contribute to the body of knowledge of systematic construction safety management in terms of the workers’ characteristics and behaviors by providing an integrated theoretical framework based on competency theory. As for the practical aspect, this study confirmed the rationality of adopting targeted strategies to optimize working performance, and also presented a tentative approach for assessing workers on sites. Furthermore, the findings also emphasized to the managers and professionals the necessity of developing training systems for making workers integrating into a crew appropriately and smoothly.

It is common for migrant workers/ethnic minorities (EMs) who are already part of society to join the construction industry. Therefore, there is a dire need to develop and utilize new management tools and strategies to accommodate the differences in language and culture of this incoming labor force [67,68]. Since the research object of this paper is the workers of Wuhan Metro Engineering, most of them are from the surrounding cities of Wuhan. In addition, due to the popularity of Mandarin, the impact of language and cultural differences has not been studied as a major influencing factor. In future research, we will focus on the multi-ethnic mixed areas in China’s border regions to understand the differences and challenges imposed by language and cultural differences.

It is notable that the proposed safety competency is just part of a worker’s overall competency, and only scaffolding workers during the metro construction process are involved in this article. A more comprehensive and more in-depth investigation containing more working trades, construction types and cultural backgrounds will be helpful to answer some unsolved issues (e.g., the ambiguous correlation of working attitudes with other latent factors), as well as to gain a profound insight into the reason for different behaviors of workers in the same working environment. Additionally, the limitations in the scope and volume of the data are expected to be addressed further by introducing a more automated and cost-efficient approach to achieve a more generalized conclusion. 

## Figures and Tables

**Table 1:** The profile of respondents.

Category	Range	Frequency	Percentage
Age	<26	15	7.3%
	26–35	36	17.6%
	36–45	65	31.7%
	46–55	69	33.7%
	>55	20	9.7%
Experience	<1	6	2.9%
	1–5	33	16.1%
	6–10	75	36.6%
	11–20	63	30.7%
	>21	28	13.7%
Education	Junior Middle School or Lower	171	83.4%
	Technical School	23	10.8%
	Senior Middle School or Higher	11	5.4%

**Table 2:** Potential items affecting workers’ safety competency.

No.	NOSSS [41]	Behavioral Event Interview	Accident Reports	Competency Dictionary [35]
1	Teamwork	“Following Standard Operation Procedure consciously.”	Good physical condition	Quality Orientation:
2	Abidance by rules	“Being familiar with the operation, and rarely violating regulations out of skills.”	Risks are not underestimated by the length of time worked or the likelihood of occurrence	Teamwork
3	Carefulness	“Dissuading the coworkers from risky behaviors.”	Self-ability and self-skills are not overestimated according to his/her own experiences	Time management
4	Agreeableness	“A strong sense of responsibility of considering the consequences of their own actions.”	Safety knowledge that ensures correct identification and disposal of risks in work.	Resilience
5	Self-Control	“Being willing to draw a lesson from others’ mistakes.”	A correct understanding of safety regulations and accidents.	Initiative
6	Communication	“Working Carefully and paying attention to details at work.”	Cautious attitudes on operation tasks.	Planning and Organizing
7	Time Management	“Cautious, won’t take risky actions by fluke.”	The strong mentality of responding to emergency calmly.	Problem Solving:
8	Responsibility	“Steady mind.”	Good adaptability of being integrated into a crew.	Communications
9	Active Protection awareness	“Being good at easing their moods.”	Teamwork	Building Trust
10	Physical quality	“Less resistance at work.”	A high level of initiative to learn skills and knowledge.	Positive working relationship
11	Legal common sense	“Rarely showing off and playing a hero during operation.”	Being able to comply with safety regulations under heavy working pressure.	Impact
12	Operation knowledge and skill	“Difficult to be impacted by the surrounded negative atmosphere.“	Qualified ability in self-control and time management.	Technical knowledge and skills
13	Learning Capacity	“Responding quickly and calmly when facing an emergency.”	Being skilled in daily operation.	Information Monitoring
14	Hazard identification	“Having a good interpersonal relationship with coworkers.”		Applied Learning
15	Logical analysis	“Good physical condition and energetic.”		Adaptability
16				Stress Tolerance
17				Following Procedures
18				Managing Conflict
19				Professional Integrity

**Table 3:** Definitions of items included in the questionnaire.

No.	Items	Definition
1	Physical Condition	Workers can consistently perform physically taxing work without losing effectiveness.
2	Professional Integrity	Workers spontaneously display and promote conduct and behaviors consistent with the fundamental organizational regulations.
3	Technical Knowledge & Skills	Workers have achieved a satisfactory level of technical and skill and knowledge in position-related areas.
4	Responsibility	Workers have a strong sense of responsibility for considering the consequences of their own actions.
5	Applied Learning	To assimilate and apply new job-related information promptly.
6	Teamwork	Workers are willing to work with and help others to accomplish task objectives
7	Time Management	High working efficiency and reasonable time arrangement.
8	Upholding the Principle	Workers are difficult to be impacted by the surrounded negative atmosphere.
9	Working Relationship	Workers intend to place a higher priority on team goals than on own purposes and gain agreement from partners to support partnership-oriented actions.
10	Prudence	Workers are able to remain cautious on operation tasks.
11	Adaptability	Workers can modify behavior to deal effectively with changes in the working environment, readily tries new approaches appropriate for the changed situation.
12	Calmness	Workers can respond to issues quickly and calmly when facing an emergency
13	Steadiness	Workers rarely try to show off or play a hero during operation.
14	Safety Awareness	Workers can realize the necessity of safety-related requirements, and identify the potentially hazardous factors affecting safety.
15	Stress Tolerance	Workers are able to maintain stable performance under pressure and handle stress in a manner that is acceptable to others.
16	Legal Common Sense	Basic legal knowledge related to occupational safety and health insurance.
17	Quality Orientation	Workers are able to accomplish tasks by considering all areas involved and show concern for quality both for the process and products.
18	Compliance with Procedure	Workers are willing to follow established procedures for completing work tasks accurately.
19	Impact	Workers dare to support professional assistance and exhibit a self-confident appearance.

**Table 4:** The statistical description of critical items affecting workers’ safety competency.

No.	Critical Items	Mean	S.D.
Item 1	Physical Condition	2.985	0.751
Item 14	Safety Awareness	2.981	0.760
Item 3	Technical knowledge and skills	2.976	0.744
Item 4	Responsibility	2.810	0.797
Item 5	Applied Learning	2.805	0.817
Item 6	Teamwork	2.717	0.821
Item 7	Time Management	2.620	0.774
Item 9	Working Relationship	2.556	0.782
Item 11	Adaptability	2.473	0.900
Item 15	Stress Tolerance	2.024	0.915
Item 8	Upholding the Principles	1.971	0.944
Item 19	Impact	1.956	0.605
Item 10	Prudence	1.927	0.960
Item 13	Steadiness	1.917	0.912
Item 2	Professional Integrity	1.893	0.979
Item 18	Compliance to Procedure	1.893	0.655
Item 12	Calmness	1.883	0.783
Item 17	Quality Orientation	1.863	0.665
Item 16	Legal Common Sense	1.434	0.658

S.D. = standardized deviation.

**Table 5:** Kaiser-Mayer-Olkin (KMO) sampling adequacy and Bartlett’s test.

**Kaiser-Meyer-Olkin Measure of Sampling Adequacy**		0.786
**Bartlett’s Test of Sphericity**	Approx. Chi-Square	960.358
	df	153
	Sig.	0.000

**Table 6:** Factor loading matrix after varimax rotation.

No.	Items	Component			
		1	2	3	4
Item 13	Steadiness	0.806	0.251		0.124
Item 2	Professional Integrity	0.856			0.219
Item 15	Stress Tolerance	0.888	0.123		0.113
Item 8	Upholding the Principles	0.796	0.133		0.124
Item 10	Prudence	0.808	0.143		
Item 4	Responsibility	0.162	0.817		0.164
Item 7	Time Management	0.191	0.699	0.166	0.169
Item 6	Teamwork	0.176	0.863		
Item 5	Applied Learning		0.780		
Item 9	Working Relationship	0.110	0.732		0.169
Item 11	Adaptability	0.282	0.764		0.158
Item 19	Impact			0.827	
Item 18	Compliance with Procedure	0.117	0.144	0.758	
Item 17	Quality Orientation			0.862	0.153
Item 14	Safety Awareness	0.138	0.313		0.744
Item 3	Technical knowledge and skills	0.153	0.154		0.758
Item 1	Physical Condition	0.158		0.121	0.779
Item 16	Calmness	0.228	0.309	0.149	0.149

**Table 7:** Cronbach’s α reliability test of identified factors.

No.	Factors	Cronbach’s α
**Factor 1**	Individual Character and Inclination (5 items)	0.889
**Factor 2**	Self-adjustment and Adaptability (6 items)	0.907
**Factor 3**	Working Attitudes (3 items)	0.758
**Factor 4**	Safety-related Operation Qualification (3 items)	0.728

**Table 8:** The convergent validity test of identified factors.

Factor	Items	FL	CR	AVE
F1. Individual Character & Inclination	Prudence	0.737	0.909	0.667
	Upholding the Principles	0.747		
	Stress Tolerance	0.917		
	Professional Integrity	0.881		
	Steadiness	0.785		
F2. Self-adjustment & Adaptability	Adaptability	0.800	0.891	0.654
	Working Relationship	0.700		
	Applied Learning	0.661		
	Teamwork	0.852		
	Time Management	0.706		
	Responsibility	0.827		
F3. Working Attitudes	Quality Orientation	0.999	0.767	0.543
	Compliance with Procedure	0.548		
	Impact	0.576		
F4. Safety-related Operation Qualification	Safety Awareness	0.806	0.778	0.540
	Technical knowledge and skills	0.658		
	Physical Condition	0.733		

FL = standardized factor loadings; CR = construct reliability; AVE = average variance extracted.

**Table 9:** Discriminant validity test of the identified factors.

Factors	F4	F3	F2	F1
F4	0.735			
F3	0.231	0.737		
F2	0.451	0.054	0.809	
F1	0.416	0.134	0.405	0.817

Figures in bold type: square root of AVE; Figures in plain type: inter-factor correlations.

**Table 10:** Hypothesis testing results.

Model	Hypothesis	Estimate	S.E.	C.R.	*p*	Results
The Original Hierarchical Model	H1. Individual Character & Inclination →Self-adjustment and Adaptability	0.454	0.079	5.769	<0.000	Adoption
	H2. Individual Character & Inclination →Working Attitudes	0.095	0.067	1.421	0.155	Rejection
	H3. Individual Character & Inclination →Safety-related Operation Qualification	0.248	0.073	3.409	<0.000	Adoption
	H4. Self-adjustment and Adaptability →Safety-related Operation Qualification	0.242	0.072	3.376	<0.000	Adoption
	H5. Working Attitudes →Safety-related Operation Qualification	0.110	0.070	1.575	0.115	Rejection
	H6. Working Attitudes →Self-adjustment and Adaptability	0.106	0.035	1.523	0.128	Rejection
The Modified Hierarchical Model	H7. Individual Character & Inclination →Safety competency	0.303	0.089	3.410	<0.000	Adoption
	H8. Self-adjustment and Adaptability →Safety competency	0.198	0.087	2.283	0.022	Adoption
	H9. Working Attitudes →Safety competency	0.305	0.089	3.424	<0.000	Adoption
	H10. Safety-related Operation Qualification →Safety competency	0.322	0.111	2.900	0.004	Adoption

Estimate = standardized regression weights; S.E. = standardized error; C.R. = critical ratio.

**Table 11:** Results of goodness-of-fit testing.

Fit indices Type	Index	Acceptable Standards	Values (Hierarchical Model)	Values (Modified Model)
Absolute fit indices	$\chi^{2}/\mathrm{df}$	<3.00 Accepted	2.631	2.326
	RMSEA	<0.09 Accepted	0.090	0.081
	GFI	>0.80 Accepted	0.866	0.867
Parsimony fit indices	PGFI	>0.50 Accepted	0.645	0.657
	PNFI	>0.50 Accepted	0.720	0.716
Incremental fit indices	CFI	>0.90 Accepted	0.906	0.908
	IFI	>0.90 Accepted	0.907	0.909

**Table 12:** Direct, indirect, and total effects of latent factors.

Factor	Relationships	Direct Effects	Indirect Effects	Total Effects
F1	Individual Character & Inclination →Safety-related Operation Qualification	0.291	0.142	0.433
	Individual Character & Inclination →Self-adjustment and Adaptability	0.448	-	0.448
	Individual Character & Inclination →Safety competency	0.428	0.336	0.764
F2	Self-adjustment and Adaptability →Safety-related Operation Qualification	0.288	-	0.288
	Self-adjustment and Adaptability →Safety competency	0.282	0.112	0.394
F3	Working attitudes →Safety competency	0.385	0.045	0.430
F4	Safety-related Operation Qualification →Safety competency	0.387	-	0.387

## Appendix A

**Table A1:** Questionnaire for construction workers’ safety competency.

**Part 1. Demographic Information (Please Fill Each Blank with the Applicable Options)**				
Name:				
Age: ( )				
A. Under 26	B. 26–35	C. 36–45	D. 46–55	F. Above 55
Experiences: ( )				
A. Less than 1 years	B. 1–5 years	C. 6–10 years	D. 11–20 years	F. Above 20 years
Education Level: ( )				
A. Junior Middle School or Lower B. Technical School C. Senior Middle School or Higher				
**Part 2. Identification of Critical Items** To grade each item with scores from 1 to 4, respectively corresponding to: 1—Strongly disagree with this assertion/My condition does not fit this statement at all. 2—Disagree with the assertion./My condition partially fits this statement. 3—Agree with the assertion./My condition mostly fits this statement. 4—Strongly agree with the assertion./My condition totally fits this statement mostly.				
**Items**	**Statement**			**Scores**
I1	I am energetic and strong enough to handle my daily work, even for the overtime task.			( )
I2	I rarely behave against the rules and regulations proposed by crew leaders.			( )
I3	I am familiar with the operating standards and able to handle the technical issues at work.			( )
I4	The errors in my own work are likely to pose a serious impact on my coworkers’ operation.			( )
I5	It does not take long for me to master an operation procedure, or get to know the changes within.			( )
I6	I prefer to work together with my colleagues, and I can be more effective then.			( )
I7	I seldom received criticism from crew leaders for being incapable of finishing tasks on work, and I am rarely distracted by irrelevant issues during operation.			( )
I8	I can adhere to the operating standards of scaffolding, even most of the coworkers that fail to meet the criterions are not punished.			( )
I9	My colleagues are willing to seek my advice, and I am able to address the conflicts within the crew without help from crew leaders.			( )
I10	During operation, any fluky actions out of convenience or other reasons are not acceptable, even for a seasoned worker.			( )
I11	It does not take long for me to modify my behavior or accept a new approach as required, and these countermeasures against changes usually work well in my experience.			( )
I12	I can respond to the emergency during operation instantly and appropriately.			( )
I13	Displaying courage during daily operation is not an appropriate way to earn other worker’s respect.			( )
I14	I think that wearing safety protection devices is useful indeed, and I am able to identify the potential risk sources in my working environment.			( )
I15	I can perform stably and safely even when facing a more intensive workload, whilst I will not ease the nervous mood in an unsuitable way. (e.g., smoking, drinking and gambling.)			( )
I16	If I get a work-related injury, I know what to do in order to guarantee my rights of claiming compensation legally. (Exclusive of asking the foreman for help.)			( )
I17	It is impossible for an operation process ignoring details to realize a set of qualified scaffolds.			( )
I18	Every single requirement in the standard operating procedure should be followed strictly for delivering a set of qualified scaffolds.			( )
I19	I am confident in my occupational skills and experiences, and I have provided technical assistance to my working team several times.			( )
